# Development of an Antimicrobial Stewardship Intervention Using a Model of Actionable Feedback

**DOI:** 10.1155/2012/150367

**Published:** 2012-02-21

**Authors:** Sameer J. Patel, Lisa Saiman, Jennifer M. Duchon, David Evans, Yu-hui Ferng, Elaine Larson

**Affiliations:** ^1^Department of Pediatrics, Columbia University, 622 W. 168th Street, PH 4W-475, New York, NY 10032, USA; ^2^Department of Infection Prevention & Control, NewYork-Presbyterian Hospital, 177 Fort Washing Avenue, Milstein 7 Center, New York, NY 10032, USA; ^3^School of Nursing, Columbia University, 617 W. 168th Street, Room 357, New York, NY, USA

## Abstract

We describe the development of an audit and feedback intervention to improve antibiotic prescribing in the neonatal intensive care unit (NICU) using a theoretical framework. Participants included attending physicians, neonatal fellows, pediatric residents, and nurse practitioners. The intervention was based on the “model of actionable feedback” which emphasizes that feedback should be timely, individualized, nonpunitive, and customized to be effective. We found that real-time feedback could not be provided for the parameters established in this study, as we had to collect and analyze numerous data elements to assess appropriate initiation and continuation of antibiotics and required longer intervals to examine trends in antibiotic use. We learned during focus groups that NICU clinicians strongly resisted assigning individual responsibility for antibiotic prescribing as they viewed this as a shared responsibility informed by each patient's laboratory data and clinical course. We were able to create a non-punitive atmosphere thanks to written informed consent from NICU attendings and assurance from leadership that prescribing practices would not be used to assess job performance. We provided customized, meaningful feedback integrating input from the participants. Adapting the principles of the “model of actionable feedback” to provide feedback for antimicrobial prescribing practices proved challenging in the NICU setting.

## 1. Introduction

Antimicrobial stewardship interventions to promote the judicious use of antibiotics have been recommended by medical organizations and governments as critical means to reduce the burden of antimicrobial resistance, cost, and toxicity [1]. The Infectious Diseases Society of America and the Society of Healthcare Epidemiology of America recommend prospective audit of antimicrobial use with interaction and feedback to the prescriber as an evidence-based component of effective antimicrobial stewardship [2]. However, audit and feedback strategies vary widely, and implementation has yielded inconsistent results [3]. This suggests that more research is needed to determine the most effective feedback interventions, including those adapted to special patient populations. Furthermore, studies of audit and feedback interventions have rarely articulated if a theoretical approach was used to guide the choice of interventions [4].

In recognition of the need for a theoretical approach, we first framed our prescriber audit and feedback intervention using the self-regulation model developed by Bandura as a component of social cognitive theory [5]. This model of change focuses on how individuals systematically make changes in their behavior to achieve a goal, with or without the help of a coach. The individual (a) chooses a goal, (b) selects and tries some strategies to reach it, (c) self-monitors to gather data to measure success, (d) makes a judgment about his/her success, and (e) experiences an increase or decrease in confidence in his/her abilities.

Like other investigators, however, we found that this and other individual behavior change theories were not consistently successful, as they needed additional constructs to account for organizational factors affecting behavior change [6]. One such theoretical construct is the “model of actionable feedback” developed by Hysong et al. [7]. This model describes the features of feedback strategies associated with high levels of adherence to clinical practice guidelines for common chronic diseases such as diabetes and hypertension in six Veterans Affairs Medical Centers. High performing institutions, defined as those with greater than median adherence to guidelines, employed feedback which exhibited the following process measure characteristics: (1) *timeliness *as monthly or more frequent feedback was associated with better performance than quarterly feedback, (2) *individualization* as individual rather than group or aggregated feedback was associated with better performance, (3) *lack of punitiveness* as staff in low-performing centers expressed concern that feedback had a punitive connotation, and (4) *customizability* as feedback deemed meaningful to recipients was associated with better performance.

A potential strength of the actionable feedback model is that it appears to bridge the gap between conceptual underpinnings and actual implementation of feedback interventions in organizational settings, particularly settings in which close cooperation between staff is essential. Thus, we used a modification of this model in a study of interdisciplinary interventions aimed to improve antibiotic prescribing in four level III NICUs affiliated with academic medical centers. While a future publication will describe the efficacy of our interventions in improving antibiotic prescribing, the goals of this paper are to describe the development and implementation of our audit and feedback intervention using a theoretical framework. We describe the challenges encountered while developing the feedback intervention for the NICU environment, the key actions undertaken to address these challenges, and early observations related to implementation of this interventions.

## 2. Methods

### 2.1. Study Design

This substudy was embedded within the Interdisciplinary NICU Antibiotic Prescribing (iNAP) Study (NINR R010821). The iNAP study goal is to improve antibiotic prescribing practices in the NICU population by studying the effects of three randomly assigned bundles of interdisciplinary interventions on antibiotic use and antimicrobial resistance. The three interdisciplinary interventions included (1) a multimodal educational program aimed to teach relevant stewardship principles from the Centers for Disease Control and Prevention (CDC) antimicrobial stewardship guidelines, (2) a computer decision support tool aimed to improve prescribing in real time for individual patients, and (3) the intervention described in this paper, that is, an audit and feedback intervention to provide NICU staff with data regarding their prescribing practices and patient outcomes. The interdisciplinary interventions were developed by the study team between September 1st 2008 and May 1st 2010.

### 2.2. Study Site and Subjects

The NICU at Morgan Stanley Children's Hospital of New York-Presbyterian at Columbia University Medical Center was randomly assigned to receive the bundle of the three interdisciplinary interventions described above. The NICU has 62 beds, with approximately 1100 discharges a year, and a mean length of stay of 22 days. Approximately 9% of the infants are <1000 grams and 22% are transferred from other institutions. Providers prescribing antibiotics include 23 neonatologists, 11 NICU fellows, 19 pediatric nurse practitioners, 7 hospitalists, and 60 pediatric residents (5 per month). Written informed consent was obtained from attending physicians for participation in the study, which was approved by the Columbia University's Institutional Review Board. Antibiotic prescribing practices were collected by trained research personnel during the baseline year (May 1st 2009 to April 30th 2010) and during the intervention year (May 1st 2010 to April 30th 2011). Data collection is ongoing for the “sustainability” year (May 1st 2011 to April 30th 2012).

### 2.3. Audit and Feedback Intervention

We performed several studies prior to commencement of the iNAP study to determine patterns of antimicrobial prescribing and identify inappropriate prescribing practices that would inform the audit and feedback intervention. First, we conducted a retrospective observational study of antibiotic use and conducted an ethnographic study of workflow in the four study NICUs [8, 9]. We also assessed neonatologists' perspectives of appropriate antimicrobial prescribing practices using vignettes derived from the observational study [10]. From our preliminary work, we learned that failure to narrow antibiotic therapy and prolonged antibiotic prophylaxis are common reasons for inappropriate antibiotic use and variation in practice. We also learned that workflow in the NICU includes frequent interruptions and that decisions about antibiotic prescribing can be discontinuous. Following randomization of the study sites to the intervention bundles, we held two focus groups to assess the attending neonatologists' perspectives about the feedback parameters and the audit and feedback process.

Based on this preparatory work, we developed 6 parameters that were utilized to audit antibiotic use (Table 1). The audit system was partially automated, but findings were reviewed by the study team to ensure accuracy. All antibiotic use was audited, and feedback related to the chosen parameters was provided to all prescribers. However, as described above, informed consent was obtained from the attending physicians, since they had ultimate responsibility for patient care.

The feedback was provided every other month during the neonatology division's morbidity and mortality conference using power point presentations. Our study team answered questions during the presentations and afterwards in face-to-face encounters and via email and telephone.

## 3. Results

The development and implementation of our audit and feedback intervention in the context of the actionable feedback model is summarized in Table 2. While the conceptual underpinnings of the theoretic model guided our approach, it was necessary to modify our feedback strategy as we encountered some challenges to implementation while adapting this model to our NICU setting. The key actions undertaken to address these challenges are described in Table 2 as well.

### 3.1. Timeliness

We found that real-time feedback could not be provided for the parameters established in this study as we had to collect and analyze numerous data elements to assess appropriate initiation and continuation of antibiotics. Secondly, we learned that because of the relatively small numbers of antibiotic courses initiated for the study parameters each month, longer intervals were required to examine trends in antibiotic use. Finally, we found that the NICU staff seemed unwilling to attend frequent presentations of the data and offered us limited opportunities to present the data at an existing meeting once every 2 months. Reasons given for unwillingness to attend more frequent presentations included clinical responsibilities and preexisting meeting commitments.

### 3.2. Individualization

We learned during focus groups that individualized feedback was not welcomed as the NICU clinicians strongly resisted assigning individual responsibility for antibiotic prescribing as they viewed this as a shared responsibility informed by each patient's laboratory data and clinical course. The NICU attending physicians described a treatment paradigm that reflected a shared, stepwise decision-making process. It was common for one prescriber to initiate therapy while another provider discontinued, continued, or modified the regimen due to short “on-service” terms and frequent cross-coverage. Clinicians expressed concerns that the feedback would incorrectly assign responsibility for inappropriate antibiotic prescribing to the wrong provider. These concerns distracted the prescribers from the educational goals of the intervention. Therefore, consistent with the preferences articulated during focus groups, we provided aggregated antimicrobial prescribing data and thus tailored the feedback according to user preferences.

### 3.3. Nonpunitive

From the study onset, we took several measures to assure prescribers that data would be kept confidential, would not be provided to the leadership, and/or would not be used to appraise their performance. NICU leadership also provided the study team with a written statement agreeing with this principle. To ensure that the neonatology attending staff understood the measures being taken to protect the confidentiality of their prescribing practices, we obtained written informed consent from them. We also obtained a certificate of confidentiality from the National Institutes of Health to further protect the data. When discussing examples of antibiotic use with the group, we did not identify individual prescribers. Finally, we were careful at all stages of the study to present a positive approach to the data audits and to emphasize the goal of improving patient safety and quality.

### 3.4. Customizability

As previously described, we created feedback parameters that were clinically meaningful to the prescribers based on preferences expressed during focus groups. We included a neonatologist on our study team and obtained ongoing support from the NICU leadership to maximize prescribers' receptiveness to the feedback intervention. We compared baseline parameters with the parameters during the intervention period to identify trends. We normalized the data using familiar units, that is, 100 patient-days. We also employed brief clinical vignettes without identifiers to describe examples of antibiotic use. As the novelty of the study waned and competing priorities vied for the NICU staff's attention, as requested, we shortened our data presentations to approximately 10 minutes.

### 3.5. Early Observations of Impact of Intervention

After being provided with data demonstrating variations in peri-operative antimicrobial prophylaxis for infants undergoing cardiac surgery, the NICU leadership formed an interdisciplinary committee to develop prophylaxis guidelines and created a method to monitor adherence to these guidelines.

## 4. Discussion

 To our knowledge, this is the first study to systematically examine the process of implementing an audit and feedback system for antibiotic prescribing. Our process included the use of the model of actionable feedback which provided a theoretical framework in which we could plan and assess our interventions and ultimately have potential explanations as to why our particular interventions did or did not work [4]. Furthermore, while planning this intervention, we recognized that behaviors in intensive care units are partly regulated by administrative or group decision making. Organizations may control behavior by punishing violations of rules and regulations rather than identifying and rewarding successes. Choices of improvement strategies may be constrained by organizational practices. Feedback may be slow because the data take time to process and feedback is usually provided to units rather than individuals.

We selected the model of Hysong et al. as they identified a group of factors that enabled organizational feedback to avoid these administrative constraints and function more like the individual feedback process described in the self-regulation model [7]. For example, timely feedback ensures that the prescriber can recall the details of the case including antibiotic use, be receptive to potential prescribing alternatives, and modify his/her behavior [11]. In our study, long durations of antibiotic courses, extensive data collection, and limited opportunities for data presentations did not allow real-time feedback as feedback was provided one to two months later. However, in this clinical scenario, this time interval may still be perceived as “timely” as the staff had recall for specific cases even weeks later, particularly for rare but potentially adverse outcomes such as inadequate treatment of a resistant organism. Alternatively, this delay may have reduced the effectiveness of our intervention for more routine clinical scenarios such as the duration of treatment for culture negative sepsis.

We were also unable to provide individualized feedback. Critical to individualization is accurate attribution of antibiotic decisions to prescribers. This task is difficult in critical care units and in settings with a large number of clinicians in training as antibiotic decision-making is shared or negotiated between different providers. Furthermore, during the focus groups, participants were concerned that one clinician would be “judged” for a previous clinician's choices and such feedback could have undermined the receptiveness of the NICU team to the feedback data. Thus, we chose to present aggregate data only. In fact, aggregate data may be preferable in a team setting such as our NICU in which a sense of collective responsibility and investment in patient safety and quality exists. Unit-level feedback may increase the receptiveness of staff and enhance a sense of communal ownership of patient safety concerns such as has been noted in strategies to improve adherence to hand hygiene and reduce central line-associated blood stream infections [12, 13].

A nonpunitive atmosphere encourages the trust of the participants and receptiveness to feedback. Several of our methods were formal (written informed consent and certificate of confidentiality at initiation of the study), which would be irrelevant in a nonresearch setting. We believe, however, that our decision to forgo individual for group feedback made it easier for the NICU staff to perceive feedback as nonpunitive. For long-term success, audit and feedback interventions not only require the support of leadership, but require a culture that encourages transparency even when adverse outcomes occur [14].

Although each prescriber could not request individual data queries, we devoted considerable effort to ensuring the customizability of our feedback intervention to make our parameters meaningful to the NICU team. Preparatory fieldwork was essential to making the intervention viable. To maximize the receptiveness of the NICU staff, we involved the NICU leadership and conducted focus groups. When presenting data at each feedback session, we reinforced study definitions for our metrics. Customizability is particularly important in a research setting where the feedback content is guided by the study protocol rather than developed by the prescribers themselves.

Our study had several limitations. The receptiveness of clinicians to specific interventions may be influenced by the leadership and social culture of the NICU. Hence, our experience may not be generalizable to other practice settings. To implement the feedback intervention, we needed the resources of the study as well as our electronic medical record system to synthesize demographic, clinical, pharmacy, and microbiological data. These resources may not be readily available in other settings.

In summary, we had only moderate success in fully incorporating the components of the model of actionable feedback in our audit and feedback intervention to improve antibiotic prescribing in the NICU. While we encountered challenges, our solutions incorporated the core principal of customizing meaningful feedback to ensure that the NICU staff would be receptive to the feedback. We believe we succeeded as evidenced by excellent participation during the feedback sessions and the crafting of guidelines for perioperative prophylaxis for cardiac surgery. A future publication will address whether our prescriber feedback intervention was successful in improving antibiotic prescribing.

## 5. Conclusions

Adapting the principles of the “model of actionable feedback” to provide feedback for antimicrobial prescribing practices proved challenging in the NICU setting. We found that real-time feedback on complex antibiotic prescribing was difficult. While a nonpunitive atmosphere was maintained, NICU clinicians strongly resisted assigning individual responsibility for antibiotic prescribing. We successfully provided customized, meaningful feedback integrating input from the participants.

## Figures and Tables

**Table 1:** Parameters of antibiotic prescribing studied in audit and feedback intervention used in the neonatal intensive care unit.

Stewardship concepts	Parameters
Inadequate coverage	(1) Empiric treatment of pathogen with ineffective antibiotic.
Appropriate diagnostic strategy	(2) Number of blood cultures obtained prior to initiation of empiric therapy for late-onset sepsis in infants with and without CVC.
Excessive antibiotics	(3) Days of treatment with broad-spectrum agent rather than narrower-spectrum agent based on pathogen susceptibilities.
	(4) Days of treatment with an agent for gram-negative pathogens following identification of a gram-positive pathogen or vice versa.
Antibiotic duration	(5) Duration of antibiotic treatment for culture-negative sepsis.
Antibiotic prophylaxis	(6) >2 antibiotic/day of perioperative antibiotics for cardiac surgery and >1 antibiotic/day for on-cardiac surgery.

**Table 2:** Components of actionable feedback for antibiotic prescribing in the NICU.

Process measures	Challenges	Key actions	Outcomes	Achieved
Timely	Prolonged data collection for prescribing practices. Limited opportunities to present data to group. Inclusion of rare outcomes.	Partially automated data analysis. Developed templates for data presentations coordinated with NICU leadership and presented data at existing meetings, for example, Morbidity and Mortality Conference emailed data to NICU prescribers prior to presentation.	After a one-month interval required to collect and analyze the data, a two-month audit of antibiotic prescribing was presented. This presentation was repeated every 2 months.	Partially

Individualized	Rotating “on-service” neonatologists with different duration of service time. Difficulty and resistance to assigning individual responsibility for specific antimicrobial usage.	Conducted focus groups with prescribers to evaluate acceptance of individual feedback.	Feedback indicated that group feedback is desired. Group feedback is provided. Deidentified examples of antibiotic use discussed.	No

Nonpunitive	Concern that results of audit would be shared with peers or used by supervisors to appraise performance.	Obtained written informed consent from neonatologists. Obtained certificate of confidentiality from National Institute for Nursing Research.	98% of eligible physicians enrolled and signed consent.	Yes

Customized	Unique patient population with limited published guidelines for appropriate antimicrobial prescribing. Different prescribing preferences among subspecialty physicians providing guidance for treatment.	Performed ethnographic studies of work flow and antibiotic decision-making using semi-structured interviews and direct observation [8]. Performed multi-center retrospective study to understand patterns of antibiotic inappropriate antimicrobial use [9]. Conducted surveys using clinical vignettes to assess prescribing preferences [10]. Conducted focus groups with prescribers to identify preferences for types of feedback.	Feedback content reflected preferences of prescribers as well as study team. Interdisciplinary committee formed to review evidence and formulate recommendations for perioperative antibiotic prophylaxis for cardiac surgery.	Yes

NICU, neonatal intensive care unit.
